# Seagrass blue carbon stocks and sequestration rates in the Colombian Caribbean

**DOI:** 10.1038/s41598-021-90544-5

**Published:** 2021-05-26

**Authors:** Oscar Serrano, Diana Isabel Gómez-López, Laura Sánchez-Valencia, Andres Acosta-Chaparro, Raul Navas-Camacho, Juan González-Corredor, Cristian Salinas, Pere Masque, Cesar A. Bernal, Núria Marbà

**Affiliations:** 1 School of Science and Centre for Marine Ecosystems Research, Edith Cowan University, Joondalup, WA Australia; 2 Centro de Estudios Avanzados de Blanes, Consejo Superior de Investigaciones Científicas, Blanes, Spain; 3 Marine and Coastal Research Institute “José Benito Vives De Andréis” INVEMAR, Calle 25 No. 2-55, Santa Marta, Colombia; 4 International Atomic Energy Agency, 98000 Principality of Monaco, Monaco; 5 Global Change Research Group, IMEDEA (CSIC-UIB), Institut Mediterrani D’Estudis Avançats, Miquel Marquès 21, 07190 Esporles, Spain

**Keywords:** Ecology, Biogeochemistry, Environmental sciences

## Abstract

Seagrass ecosystems rank amongst the most efficient natural carbon sinks on earth, sequestering CO_2_ through photosynthesis and storing organic carbon (C_org_) underneath their soils for millennia and thereby, mitigating climate change. However, estimates of C_org_ stocks and accumulation rates in seagrass meadows (blue carbon) are restricted to few regions, and further information on spatial variability is required to derive robust global estimates. Here we studied soil C_org_ stocks and accumulation rates in seagrass meadows across the Colombian Caribbean. We estimated that *Thalassia testudinum* meadows store 241 ± 118 Mg C_org_ ha^−1^ (mean ± SD) in the top 1 m-thick soils, accumulated at rates of 122 ± 62 and 15 ± 7 g C_org_ m^−2^ year^−1^ over the last ~ 70 years and up to 2000 years, respectively. The tropical climate of the Caribbean Sea and associated sediment run-off, together with the relatively high primary production of *T. testudinum*, influencing biotic and abiotic drivers of C_org_ storage linked to seagrass and soil respiration rates, explains their relatively high C_org_ stocks and accumulation rates when compared to other meadows globally. Differences in soil C_org_ storage among Colombian Caribbean regions are largely linked to differences in the relative contribution of C_org_ sources to the soil C_org_ pool (seagrass, algae *Halimeda tuna*, mangrove and seston) and the content of soil particles < 0.016 mm binding C_org_ and enhancing its preservation. Despite the moderate areal extent of *T. testudinum* in the Colombian Caribbean (661 km^2^), it sequesters around 0.3 Tg CO_2_ year^−1^, which is equivalent to ~ 0.4% of CO_2_ emissions from fossil fuels in Colombia. This study adds data from a new region to a growing dataset on seagrass blue carbon and further explores differences in meadow C_org_ storage based on biotic and abiotic environmental factors, while providing the basis for the implementation of seagrass blue carbon strategies in Colombia.

## Introduction

Seagrass meadows rank amongst the most prized ecosystems on Earth due to the provision of vital ecosystem services such as nutrient cycling, biodiversity and contribution to climate change mitigation and adaption through carbon sequestration and coastal protection despite they occupy less than 0.1% of the global marine surface^1,2^. Facing the urgency to reduce atmospheric greenhouse gases to mitigate climate change, the strategy known as blue carbon builds up on the conservation and restoration of coastal vegetated ecosystems (mangrove forests, tidal marshes, seagrass meadows and macroalgae beds) to enhance and preserve their capacity to act as natural carbon sinks^3^. It is estimated that seagrass globally store 140 Mg organic carbon (C_org_) per hectare in the top meter of soils, accumulated over centennial-millenial time scales, being up to 40 times more efficient at capturing organic carbon (C_org_) than land forests soils^4,5^.

Despite those benefits and services provided, seagrasses are among the most threatened ecosystems on the planet, and widespread die-off of seagrasses has been estimated at 0.9% year^−1^, due to a diversity of pressures including coastal development and coupled nutrient and sediment discharges causing eutrophication and siltation, respectively^6^. More recently, local conservation and management actions resulted in the deceleration and reversal of seagrass declining trends in Europe^7^ and USA^8^. However, it has been estimated that 0.15 Pg of CO_2_ may be released annually from disturbed seagrass ecosystems, which is equivalent to 3% of those from deforestation globally^9^. The inclusion of seagrass conservation and restoration projects into carbon crediting markets could provide a financial incentive to preserve the ecosystem services they provide, including their role in C_org_ storage and climate change mitigation and adaptation^10^.

Seagrass meadows are found along the shores of all continents except Antarctica occupying a global area estimated to range between 0.27 to 1.65 million^2,11,12^ km^2^, and encompass about 70 seagrass species^13^. Seagrass species have a broad dissimilarity in traits including differences in biomass and primary production rates^14^, that also vary across environmental conditions such as water depth and geomorphology^15,16^. Such differences in biotic and abiotic habitat characteristics result in up to 18-fold variability in C_org_ storage capacity across seagrass ecosystems^17^. An increasing number of studies are reporting seagrass blue carbon stocks and accumulation rates at local, regional and global scales^5,18–23^. However, there is a scarcity of seagrass blue carbon estimates in American countries, with the exception of Mexico, Canada, the United States in North America, and a few studies in Central and South America (Brazil, Panama and Dutch Caribbean)^5,20,23–29^. The scarcity of seagrass blue carbon studies in these key regions is limiting our capacity to derive robust global estimates while precluding their incorporation into national carbon accounting and the implementation of blue carbon strategies within Nationally Declared Contributions to mitigate climate change.

The Caribbean Sea, a tropical region with climates ranging from semi-arid to rain forests, provides a case for a region which is currently not represented in global estimates of seagrass blue carbon. Seagrasses grow in reef lagoons between the beaches and coral reefs in the Caribbean, and can form extensive meadows in protected embayment’s and estuaries. Out of the seven seagrass species found within the Caribbean Sea, *Thalassia testudinum* (turtle grass) is the most abundant species and forms persistent and climax seagrass ecosystems in the region^13^. The current area of *T. testudinum* seagrass beds in the Colombian Caribbean has been estimated in 661 km^2^, but extensive losses have also been documented^30^. For instance, Cartagena Bay experienced more than 90% reduction in seagrass extent over the past decades (from 2.5 to 0.18 km^2^) attributed to local anthropogenic disturbances^31^. Past and current anthropogenic threats to seagrass in the Caribbean region include coastal development, mining, sediment run-off and pollution, while natural threats are mainly related to hurricanes^32^.

**Figure 1:**
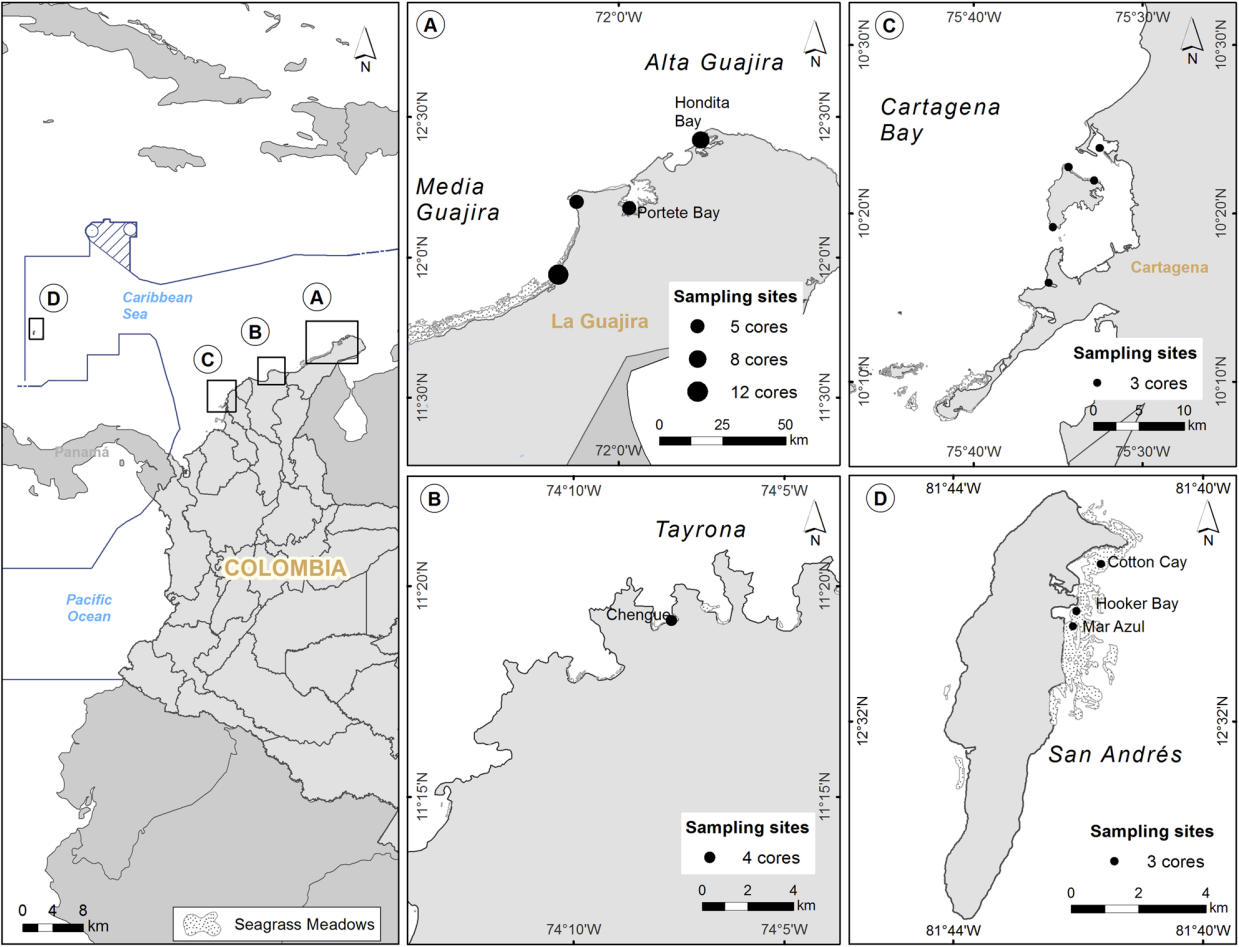
Seagrass meadow distribution and location of the sampling sites in the Colombian Caribbean. Left panel shows the location of the five regions studied (Alta Guajira, Media Guajira, Cartagena Bay, Tayrona and San Andrés). (**A**–**D**) show the sampling sites within each region. The size of the circles indicates the number of cores studied at each sampling site. The maps were created in ArcGis Online (https://www.esri.com/en-us/arcgis/products/arcgis-online/overview).

This study aims to provide estimates of seagrass soil C_org_ stocks and accumulation rates for the underrepresented Colombian Caribbean (Fig. 1), based on biotic and abiotic environmental factors interacting at regional scales. We combine estimates of C_org_ density in 1 m-thick soils from *T. testudinum* meadows with soil accumulation rates derived from sediment chronologies determined with ^210^Pb and ^14^C to estimate soil C_org_ stocks within the top meter of soil and short- (last 70 years) and long-term (last 2000 years) soil C_org_ accumulation rates. We also assess differences in soil C_org_ storage among habitats based on the contribution of seagrass-C_org_ and allochthonous-C_org_ sources to the soil C_org_ pool, and sediment grain-size. The information gathered provides baseline values for future development of blue carbon strategies in Colombia as well as key information for regional and national marine planning and governance based on seagrass blue carbon ecosystem service.

## Results

Compression of seagrass soils during coring operations averaged 37 ± 16% (mean ± SD; Supplementary Information Table A). The top meter of seagrass soils of the Colombian Caribbean had a mean ± SD dry bulk density (DBD) of 0.87 ± 0.32 g cm^−3^, containing 6.6 ± 4.0% organic matter (OM), 1.2 ± 0.9% organic carbon (C_org_) and 7.9 ± 3.5 mg C_org_ cm^−3^ (Table 1). Seagrass soils were mainly constituted of particles > 0.016 < 0.125 mm (33 ± 24%), with a relatively high abundance of particles < 0.016 mm (11 ± 14%) and coarse sands > 0.5 mm (21 ± 21%). Soil C_org_ stocks in the top first meter ranged from 39 to 673 Mg C_org_ ha^−1^ (241 ± 118 Mg C_org_ ha^−1^; Table 1).

**Table 1:** Mean (± SD) dry bulk density (g cm^−3^), organic carbon content (C_org_, %), organic matter content (OM, %), δ^13^C (‰), sediment grain-size < 0.016 mm, > 0.0.016 < 0.125 mm and > 0.5 mm (%) and soil organic carbon stocks (in 1 m-thick, kg C_org_ m^−2^) in seagrass soils from all cores studied in the different locations and regions in the Colombian Caribbean.

Region	Location	Core ID	DBD (g cm^−3^)		C_org_ (%)		OM (%)		δ^13^C (‰)		< 0.016 mm (%)		> 0.016 < 0.125 mm (%)		> 0.5 mm		Soil C_org_ stock in 1 m-thick
			Mean	SD	Mean	SD	Mean	SD	Mean	SD	Mean	SD	Mean	SD	Mean	SD	(Mg C_org_ ha^−1^)
Alta Guajira	Cabo de la Vela	AGCV1	1.0	0.2			5.0	0.8									224
		AGCV2	1.0	0.0			4.9	0.7									246
		AGCV3	0.9	0.0			4.2	0.6									203
		AGCV4	1.1	0.1			4.5	1.8									279
		AGCV5	0.9	0.0			6.2	0.9									329
	Portete Bay	AGBP1	0.6	0.2			16.8	5.2									673
		AGBP2	0.4	0.2			14.8	4.7									344
		AGBP3	0.7	0.1			10.7	5.0									470
		AGBP4	0.6	0.1			10.3	2.8									369
		AGBP5	0.7	0.1			5.7	0.1									235
	Hondita Bay	AGBH1	0.8	0.0			10.3	3.6									415
		AGBH2	0.8	0.1			8.4	3.0									370
		AGBH3	0.8	0.3	1.3	1.1	8.9	3.2	− 21.4	1.3	16.7	5.7	41.5	12.5	11.8	14.9	266
		AGBH4	0.9	0.2			10.8	4.1									479
		AGBH5	0.8	0.2			8.2	1.7									381
		AGBH6	0.5	0.1	1.8	0.9	11.6	3.4	− 20.3	2.0	45.6	2.0	52.0	1.9	0.0	0.0	206
		AGBH7	0.5	0.0			12.1	2.6									350
		AGBH8	0.6	0.1			15.9	3.5									522
Media Guajira	Carrizal	MGCZ1	1.2	0.1			3.3	2.0									151
		MGCZ2	1.1	0.1			6.5	1.8									357
		MGCZ3	1.1	0.3			5.2	1.2									258
		MGCZ4	1.1	0.2	0.5	0.1	2.4	0.7	− 21.5	0.6	4.8	0.1	64.9	1.9	1.2	2.3	108
		MGCZ5	1.1	0.1			2.9	0.6									153
		MGCZ6	1.0	0.1			3.5	1.2									159
		MGCZ7	0.9	0.2			3.6	4.1									130
		MGCZ8	1.0	0.2			5.7	3.9									203
		MGCZ9	1.1	0.1			5.3	0.9									252
		MGCZ10	1.0	0.3	0.5	0.2	3.6	0.6	− 20.0	0.9	3.4	1.7	71.0	18.3	2.3	6.4	145
		MGCZ11	1.0	0.0			5.6	2.5									309
		MGCZ12	1.1	0.1			4.7	3.6									267
	Musichi	MGMC1	1.2	0.3	0.6	0.1	3.2	1.9	− 22.8	1.0	3.5	0.8	64.4	21.1	6.8	10.0	139
		MGMC2	1.3	0.1			2.9	0.9									176
		MGMC3	1.1	0.3			5.2	2.6									338
San Andrés	Mar Azul	SAMA1	0.6	0.1	1.5	0.5	7.6	2.8	− 16.5	1.0	23.5	3.0	35.9	4.1	0.0	0.0	193
		SAMA2	0.7	0.1			7.5	0.9									251
		SAMA3	0.7	0.1			5.9	0.8									209
	Hooker Bay	SABH1	0.6	0.2	1.7	0.9	8.2	1.7	− 15.3	1.4	10.8	3.6	33.4	6.7	17.6	11.3	206
		SABH2	0.8	0.2			7.4	3.2									258
		SABH3	0.8	0.2			5.6	4.0									179
	Cotton Cay	SACC1	0.9	0.2	1.1	0.8	4.9	1.6	− 17.2	1.1	5.7	5.9	15.6	11.9	41.9	19.4	197
		SACC2	1.0	0.1			6.5	1.2									331
		SACC3	1.1	0.1			5.9	1.1									343
Tayrona	Chengue Bay	SMCH1	0.7	0.1			6.4	3.1									217
		SMCH2	1.0	0.1			5.0	1.4									229
		SMCH3	1.2	0.1			5.2	1.5									240
		SMCH4	1.2	0.1			5.0	0.9									238
Cartagena	La Virgen	CLV1	0.8	0.0			5.2	0.0			2.4	0.9	11.5	1.1	44.4	8.4	148
		CLV2	0.8	0.1			7.1	3.2			3.1	1.1	12.1	1.1	38.1	10.6	219
		CLV3	0.7	0.0			5.2	0.3			1.6	1.0	10.0	2.1	43.6	10.0	138
	Tierra Bomba	CTTB1	0.9	0.1			5.3	0.9			8.5	5.0	28.4	14.8	25.6	12.9	168
		CTTB2	0.9	0.0			5.8	0.8			9.5	4.8	25.8	13.3	29.8	10.7	184
		CTTB3	1.0	0.0			4.8	1.2			6.6	5.2	21.1	16.6	28.6	16.7	162
	Punta Arena	CTPA1	0.8	0.0			4.1	0.3			0.2	0.5	6.8	3.8	47.6	7.7	119
		CTPA2	1.1	0.0			4.4	0.3			0.9	1.2	8.6	4.4	42.8	8.4	159
		CTPA3	0.9	0.0			4.2	0.3			0.9	1.2	8.2	3.4	44.8	2.8	142
	Cocon Bay	CTBC1	0.9	0.1			4.5	0.8			0.0	0.0	2.7	3.2	62.9	6.7	141
		CTBC2	1.2	0.1			4.6	0.2			0.4	0.6	6.7	1.9	45.3	14.5	200
		CTBC3	1.4	0.0			4.4	0.8			0.5	0.5	8.2	4.2	59.0	12.5	226
	Bocagrande	CTBG1	1.3	0.0			1.4	0.2			0.0	0.0	6.4	1.4	33.0	8.3	39
		CTBG2	1.3	0.1			1.3	0.1			0.0	0.0	8.1	2.3	23.3	10.8	40
		CTBG3	1.9	0.5			1.0	0.2			0.0	0.0	5.1	2.9	31.0	6.5	40
OVERALL			0.9	0.3	1.2	0.9	6.6	4.0	− 19.4	2.8	11.1	13.7	33.5	23.9	21.2	21.5	241 ± 117

A total of 11 cores were processed to determine the sedimentation rates during the last decades using the ^210^Pb dating method. Due to the high degree of mixing presented in five of the cores analyzed, it was only possible to determine sediment accumulation rates in six cores. It was possible to estimate sedimentation rates during the last 500–2000 years using the ^14^C dating method in five cores, based on one to three radiocarbon results per core (Supplementary Information Table B). Short- and long-term soil accretion rates (SAR) ranged from 0.9 to 7.0 mm year^−1^ (3.9 ± 0.4 mm year^−1^) and 0.37 to 3.4 mm year^−1^ (1.8 ± 0.9 mm year^−1^), respectively, while short- and long-term C_org_ accumulation rates (CAR) ranged from 34 to 195 g C_org_ m^−2^ year^−1^ (122 ± 62 g C_org_ m^−2^ year^−1^) and from 2.2 to 27.5 g C_org_ m^−2^ year^−1^ (14.9 ± 7.2 g C_org_ m^−2^ year^−1^), respectively (Table 2).

**Table 2:** Mean (± SD) short-term (last decades) and long-term (last 2000 years) sediment accumulation rates (SAR; mm year^−1^) and organic carbon accumulation rates (CAR; g C_org_ m^−2^ year^−1^) in seagrass soils from all cores successfully dated in the different locations and regions in the Colombian Caribbean.

Region	Location	Core ID	Short-term SAR	Long-term SAR	Short-term CAR	Long-term CAR
			(mm year^−1^)	(mm year^−1^)	(g C_org_ m^−2^ year^−1^)	(g C_org_ m^−2^ year^−1^)
Alta Guajira	Bahia Portete	AGBP2	1.8 ± 0.3		93.3 ± 17.1	
	Bahia Hondita	AGBH3	0.89 ± 0.16	0.44 ± 0.09	33.6 ± 5.9	4.5 ± 0.9
Media Guajira	Carrizal	MGCZ3	3.3 ± 0.8		79.5 ± 20.1	
	Musichi	MGMC1		0.37 ± 0.17		2.20 ± 1.01
San Andrés	Mar Azul	SAMA1	6.97 ± 0.61	3.36 ± 1.35	167 ± 15	27.5 ± 11.1
	Bahia Hooker	SABH1	5.97 ± 0.28	2.14 ± 1.67	164 ± 8	19.1 ± 14.9
	Cotton Cay	SACC1	4.55 ± 0.15	2.84 ± 1.11	195	21.2 ± 8.2
OVERALL			3.9 ± 0.4	1.8 ± 0.9	122 ± 62	14.9 ± 7.2

**Figure 2:**
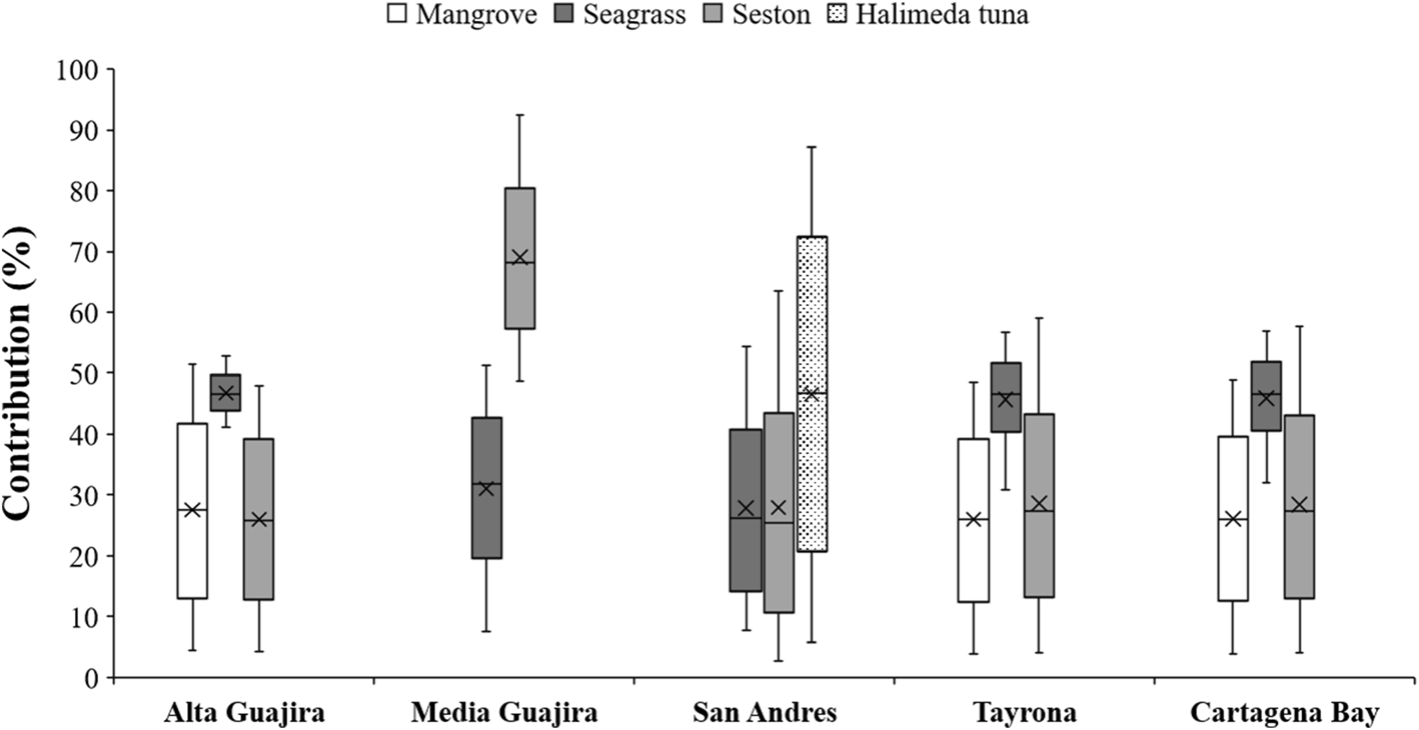
Box plots showing the results of the isotope mixing models to determine the contribution of sources (seagrass, seston, *Halimeda tuna* and mangrove) to the soil organic carbon pool in the five study sites.

**Figure 3:**
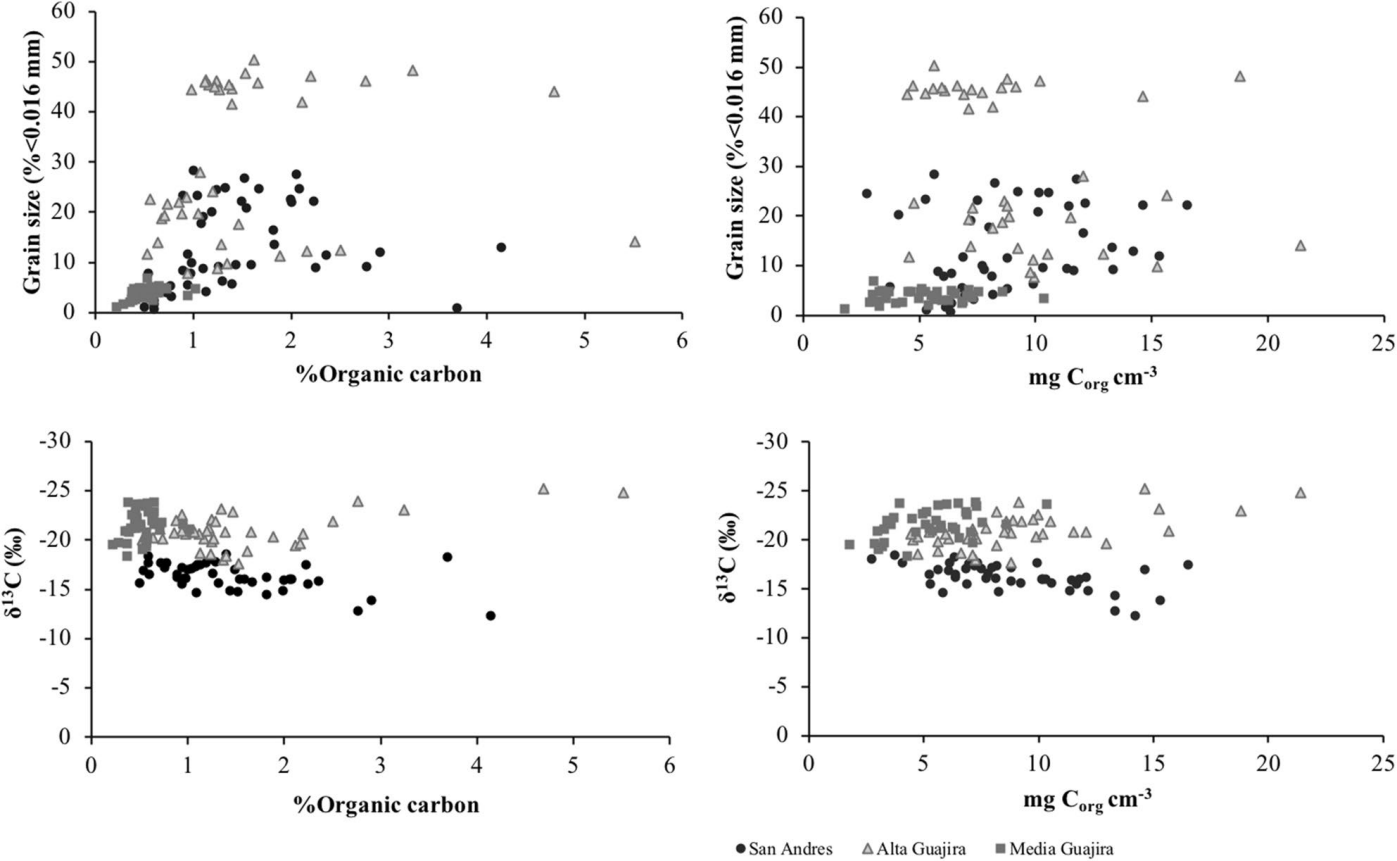
Relationships between soil organic carbon content (% C_org_ and mg C_org_ cm^−3^) and sediment particles < 0.016 mm and δ^13^C (‰) in the seagrass cores from San Andrés, Cartagena Bay and La Guajira in the Colombian Caribbean.

The δ^13^C values of sedimentary organic C_org_ in seagrass soils averaged -19.4 ± 2.8‰. The δ^13^C values of seagrass, mangrove, seston and *Halimeda tuna* (Supplementary Information Table C) were used to run mixing models to estimate the contribution of potential sources into the sedimentary C_org_ pool. Seagrass detritus and seston were the most important source of C_org_ in seagrass soils from the Colombian Caribbean (39 ± 13% and 36 ± 24%, respectively), with the exception of meadows at San Andrés where macroalgae *H. tuna* contributed 47 ± 29%. Mangrove matter contributed 27 ± 15% across Alta Guajira, Tayrona and Cartagena Bay regions where mangroves are present (Fig. 2). Overall, C_org_ (% and mg C_org_ cm^−3^) contents increased with increasing fine particle contents (% < 0.016 mm) in all regions except Alta Guajira (Fig. 3). There was a lack of relationship between δ^13^C and C_org_ (% and mg C_org_ cm^−3^) contents.

The DBD increased with soil depth in the seagrass meadows at Alta Guajira, Media Guajira and San Andrés, but remained relatively stable at Tayrona and Cartagena Bay (Supplementary Information Figure A). OM and C_org_ contents decreased with soil depth at all sites, while δ^13^C remained stable with soil depth. The content of particles < 0.016 mm remained relatively stable along soil depth, with the exception of higher content below 100 cm depth at Cartagena Bay. Coarse sand (> 0.5 mm) content was relatively higher within the upper 20 to 90 cm at Alta Guajira, Media Guajira and Cartagena Bay, while oscillated between 5 to 40% along the soil cores at San Andrés.

**Figure 4:**
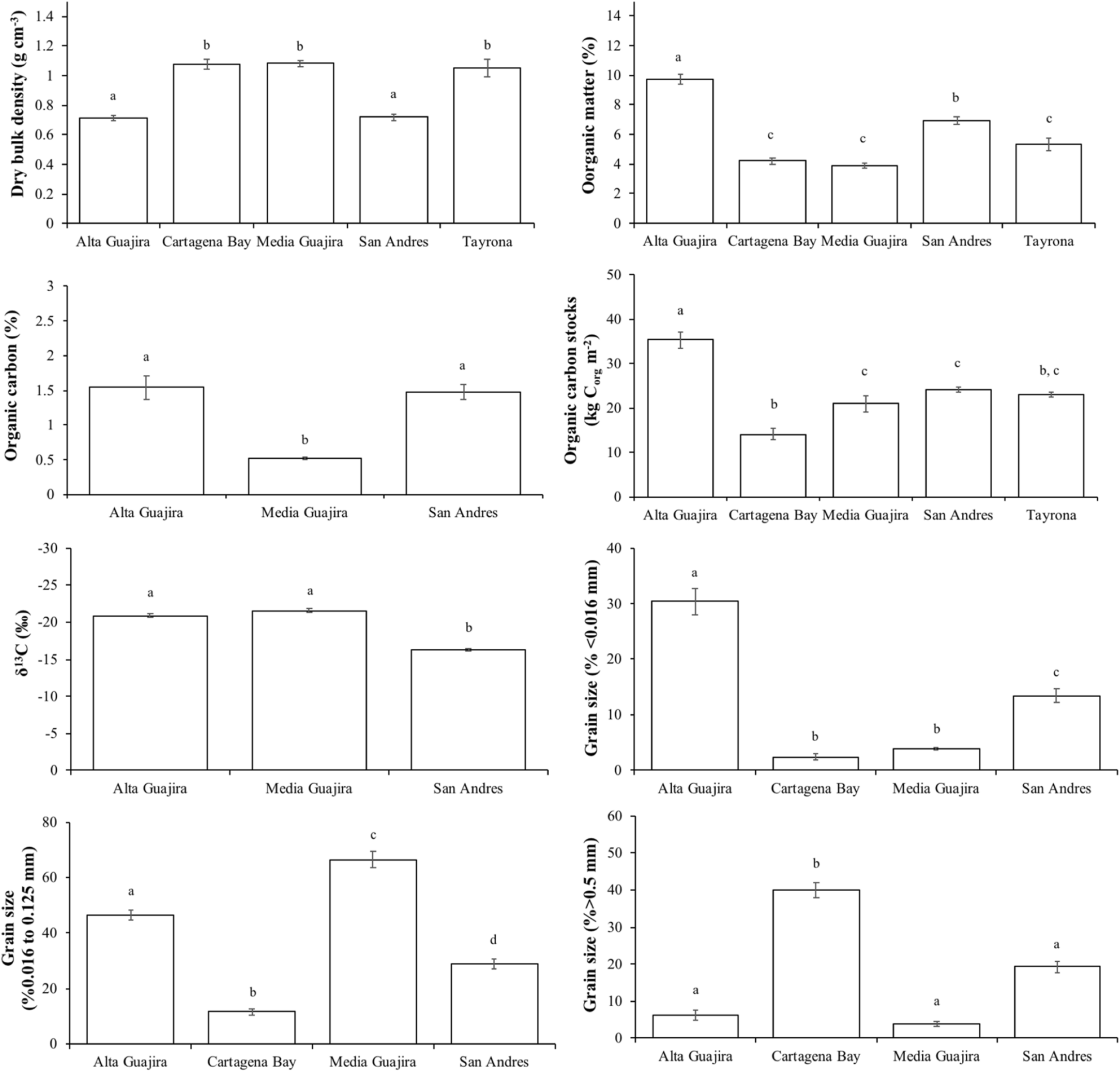
Mean (± SE) dry bulk density (g cm^−3^), organic matter (%), organic carbon (% C_org_ and kg C_org_ m^−2^ in 1 m-thick soils), δ^13^C (‰), and sediment grain-sizes < 0.016 mm, > 0.0.016 < 0.125 mm and > 0.5 mm (%) in seagrass soils from Alta Guajira, Cartagena Bay, Media Guajira and San Andrés in the Colombian Caribbean. Results of GLM tests to assess differences are denoted: different letters (a, b, c) indicate significant differences (P < 0.05).

Soil biogeochemical characteristics (DBD, %C_org_, %OM, δ^13^C, and particles < 0.016 mm, > 0.016 < 0.125 mm and > 0.5 mm) and soil C_org_ stocks were significantly different (P < 0.05) among regions (Alta Guajira, Media Guajira, Tayrona, Cartagena Bay and San Andrés; Table 3). Soil DBD in meadows at Alta Guajira (0.7 ± 0.3 g cm^−3^) and San Andrés (0.7 ± 0.2 g cm^−3^) were significantly lower than at the other regions (average ranging from 1.0 to 1.1 g cm^−3^; Fig. 4). The soil OM content was significantly higher in Alta Guajira (9.7 ± 4.6% OM) compared to meadows at San Andrés (6.9 ± 2.5% OM), while OM content at both Alta Guajira and San Andrés was higher than at Media Guajira, Tayrona and Cartagena Bay (ranging from 3.9 to 5.3%; Table 3). The soil C_org_ content followed a similar pattern, with significantly higher values at Alta Guajira (1.54 ± 1.02% C_org_) and San Andrés (1.47 ± 0.80% C_org_) compared to Media Guajira (0.52 ± 0.16% C_org_). The C_org_ stocks within the top meter of soil were up to twofold higher in Alta Guajira (353 ± 125 Mg C_org_ ha^−1^) compared to Cartagena Bay (142 ± 61 Mg C_org_ ha^−1^), while the other regions had intermediate soil C_org_ stocks with average values ranging from 210 to 241 Mg C_org_ ha^−1^. The lower number of SAR and CAR data across regions precluded comparisons. The δ^13^C values of soil C_org_ were significantly lower in Alta and Media Guajira (average ranging from -21 to -22‰) than in San Andrés (-16 ± 1.4‰). Based on isotope mixing models, seagrass was the major contributor to the soil C_org_ pool in the meadows located at Alta Guajira, Tayrona and Cartagena Bay (46% in all cases), with ~ 25% contributions of each seston and mangrove matter (Fig. 2). At Media Guajira, seston contributed 69%, while seagrass contributed the remaining 31%. The contribution of *H. tuna* to soil C_org_ pool at San Andrés was estimated at 47%, with seagrass and seston contributing 27% each. Seagrass soils at Alta Guajira had significantly higher amount of particles < 0.016 mm (30 ± 15%) than the other regions (ranging from 2 to 13%), while particles > 0.016 < 0125 mm were more abundant in Media Guajira (67 ± 17%) and Alta Guajira (46 ± 11%) (ranging from 12 to 29%; Table 1). The concentration of coarse sands > 0.5 mm was significantly higher in Cartagena Bay (40 ± 15%) compared to the other regions (ranging from 4 to 19%).

**Table 3:** Results of the Generalized Linear Models (GLM) testing for significant effects of region (Alta Guajira, Media Guajira, San Andrés, Tayrona, Cartagena) on seagrass soil dry bulk density (g cm^−3^), soil organic carbon content (%), soil organic matter content (%), soil organic carbon stocks (Mg C_org_ ha^−1^ in 1-m thick) , δ^13^C (‰), and content of sediment grain-sizes < 0.016 mm, > 0.0.016 < 0.125 mm and > 0.5 mm (%) in the Colombian Caribbean.

Variable	Factor	F	df	*P* value
Dry bulk density (g cm^−3^)	Region	74.23	4	< 0.001
Organic carbon (%)	Region	21.25	2	< 0.001
Organic matter (%)	Region	83.73	4	< 0.001
C_org_ stocks (Mg C_org_ ha^−1^)	Region	12.47	4	< 0.001
δ^13^C (‰)	Region	148.94	2	< 0.001
< 0.016 mm (%)	Region	93.36	3	< 0.001
> 0.016 < 0.125 mm (%)	Region	180.86	3	< 0.001
> 0.5 mm (%)	Region	145.13	3	< 0.001

## Discussion

Seagrasses are sparsely distributed along the Colombian Caribbean, but their C_org_ stocks in 1 m-thick soils (241 Mg C_org_ ha^−1^ on average) are well above the values from global estimates (140 Mg C_org_ ha^−1^)^5^ and other tropical regions such as Mexico, US and Indonesia (ranging^23,33–35^ from 71 to 170 Mg C_org_ ha^−1^. Estimates of soil short-term CAR (122 g C_org_ m^−2^ year^−1^) in Colombian seagrass meadows is higher than in the Dutch Caribbean (84 g C_org_ m^−2^ year^−1^)^25^ and Australian (36 g C_org_ m^−2^ year^−1^)^21^ meadows. The relatively high C_org_ sink capacity of *Thalassia* seagrass meadows in Colombia could be explained by a combination of biogeochemical and environmental processes linked to habitat characteristics. The up to twofold higher above- and below-ground biomass and production of *T. testudinum* compared to the mean of seagrass species globally^14^, likely provides a relatively larger amount of seagrass detritus for burial. Indeed, the tropical wet climate in Colombia together with the numerous rivers discharging along the coastline results in the accumulation of fine-sized particles < 0.0125 mm (44 ± 3%) within seagrass meadows (Fig. 4), which have been shown to be conductive of soil C_org_ preservation^36,37^. In turn, terrestrial run-off and mangrove vegetation deliver allochthonous C_org_ into the coastal zone that contributes to the seagrass soil carbon pool (54 ± 0.1%)^38^. SAR in Colombian seagrass meadows averaged 3.9 ± 0.4 mm year^−1^ over the last century, which are up to fourfold higher than previously reported for seagrass meadows globally^1^, and for China, Japan, Mediterranean Sea, Arabian Gulf and Australia (ranging from 0.8^19,21,39^ to 2.1 mm year^−1^, but similar to *Zostera marina* meadows^20^. High SAR entails rapid burial of organic matter in anoxic conditions thereby reducing C_org_ remineralization rates and enhancing C_org_ storage^15^. The lack of excess ^210^Pb in some of the cores analysed suggests that there is no net accumulation in some areas, or accumulation of reworked materials. Further studies (e.g. deployment of surface elevation tables to measure sediment accretion in situ) are required to overcome the limitations of radioisotopes^40^. Overall, the high seagrass primary production together with the rapid accumulation of suspended particles results in highly depositional environments that enhance carbon accumulation and preservation, which could explain the relatively high C_org_ storage capacity of seagrass meadows along the Colombian Caribbean compared to other meadows globally.

### Differences in seagrass soil carbon storage across Colombian regions

The up to threefold variability in seagrass soil C_org_ stocks among regions in the Colombian Caribbean appears to be largely linked to the relative contribution of C_org_ sources to the soil C_org_ pool (seagrass, algae, mangrove and seston) and inputs of fine sediment particles binding C_org_ and enhancing its preservation. The limited number of cores that were successfully dated with ^210^Pb and/or ^14^C (N = 6 each) precluded comparison of CAR among regions.

La Guajira contains 88% of the country's *T. testudinum* seagrass extent (581 km^2^) and seagrass soil C_org_ stocks (12.4 Tg C_org_). Although Alta Guajira only contains 18 km^2^ of seagrass meadows, it holds the largest soil C_org_ stocks per unit area in Colombia (352 Mg C_org_ ha^−1^)^41^, which also rank among the highest values reported globally^5^. The high soil C_org_ stocks per unit area in Alta Guajira are likely related to the high depositional nature of their habitats, restricted to enclosed embayments protected from hydrodynamic energy and hurricanes, with the exception of the meadows located in Cabo de la Vela^42^. The seagrass soil in Alta Guajira are mainly composed of soil inorganic particles < 0.125 mm (76%), which together with the contribution of mangrove and seston matter to the soil C_org_ pool (28% and 26%, respectively) likely led to enhanced soil C_org_ accumulation and preservation in seagrass meadows (Figs. 2 and 4). The lack of correlation between particles < 0.016 mm and C_org_ content in Alta Guajira can be explained by the saturation of particles with adsorbed organic C_org_ together with the presence of coarse seagrass detritus^37^. The Alta Guajira region is exposed to extreme climate events driven by tropical storms and hurricanes, which can result in the sudden destruction of seagrass meadows^41,42^ and the erosion of seagrass soil C_org_ stocks^43^. Since the onset of seagrass monitoring at Cabo de la Vela in 2015, one cyclone disruption event resulted in the erosion of > 0.5 ha of seagrass meadows and the top ~ 0.6 m of soils underneath the meadows^41^, likely resulting in CO_2_ emissions from perturbed soil C_org_ stocks. However, seagrasses in the protected embayments of Hondita Bay and Portete Bay within Alta Guajira remained undisturbed^41^. Further studies are required to assess the risk of tropical storms for blue carbon projects in tropical regions, in particular in the face of increased frequency and intensity of extreme climate events under predicted climate change scenarios^44^.

The relatively low seagrass soil C_org_ stocks in Media Guajira (210 Mg C_org_ ha^−1^) compared to the Alta Guajira (352 Mg C_org_ ha^−1^) is likely related to the severe exposure of meadows to hydrodynamic energy in the former. This region is constantly exposed to the currents and swell generated by strong winds from the East and Northeast, which results in the resuspension of particulate materials and turbid waters that limit the water depth limit for seagrass to up to 7 m. These processes are reflected in the relatively low content of sediment particles < 0.016 mm (4%) and dominance of particles > 0.016 < 0.125 mm (66%), which together with the low seagrass contribution to the soil C_org_ pool (31%) compared to seston (69%) suggests that meadows in this region are thriving in a highly dynamic environment compared to other Colombian Caribbean regions^42^ that could be limiting their productivity owing to reduced irradiance.

Meadows along the rocky coastline of Tayrona are restricted to enclosed environments protected from the swell (only 0.9 km^2^ of meadows mapped)^31^. The soil C_org_ stocks at our study site were relatively high (231 Mg C_org_ ha^−1^), likely related to the high productivity of the meadows thriving in clear waters, and the input of seston and mangrove detritus from adjacent creeks. However, the lack of stable C_org_ isotope and grain size analyses in the cores from this region preclude further interpretations.

Seagrass meadows have been severely decimated at Cartagena Bay over the past decades (from 100 km^2^ in 1930s to 9 km^2^ in 2001^45^. In turn, only small patches of seagrass meadows (< 0.1 km^2^ each) remain at Cartagena Bay, and their soil C_org_ stocks are the lowest found across Colombia (142 Mg C_org_ ha^−1^). Previous studies showed that soil C_org_ stocks are lower in patchy seagrass landscapes compared to continuous and extensive meadows^36,46^. Indeed, coarse sand and gravel (40%) originated from adjacent corals dominate the soil inorganic particles in Cartagena Bay’ meadows, which together with the high degree of anthropization in the region could explain the weakening of seagrass C_org_ sinks in this region^47^.

Seagrass meadows in San Andrés and Providencia Archipelago (5 km^2^) are located in a coastal lagoon basin protected from currents and waves, and to a lesser degree from tropical storms and cyclones by a reef barrier, which facilitates sedimentation^48,49^ and likely explains the relatively high soil C_org_ stocks in this region (241 Mg C_org_ ha^−1^). The short-term CAR reported for this region (175 g C_org_ m^−2^ year^−1^) are the highest reported to date for seagrass meadows, with the exception of two *P. oceanica* meadows from the Mediterranean Sea (ranging from 202 to 249 g C_org_ m^−2^ year^−1^)^50^. The seagrass soils in San Andrés contain large amounts of *H. tuna* inorganic and organic (47%) matter. The calcareous macroalgae *Halimeda* spp. is common in coral reefs and has very high primary production rates^51,52^ that likely contribute to enhance SAR through high carbonate production, and CAR through rapid burial of C_org_ in anoxic conditions. Further studies are required to understand the specific role of *H. tuna* in blue carbon, including the implications of calcification and dissolution in the carbon cycle^53^ as well as those enhancing CAR.

### Seagrass blue carbon in the Colombian Caribbean

Seagrass *T. testidinum* in the Caribbean region of Colombia occupies 661 km^2^, with 18 km^2^ in Alta Guajira, 563 km^2^ in Media and Baja Guajira, 0.9 km^2^ in Tayrona, 9 km^2^ in Cartagena Bay, 5 km^2^ in San Andrés and surrounding islands, and 65 km^2^ in the Caribbean coastline west of Cartagena Bay^45^. Based on the spatially-explicit estimates of seagrass extent and soil C_org_ stocks and accumulation rates in the five regions studied (Fig. 4), we estimate that seagrass in the Colombian Caribbean contain 14 Tg of C_org_ in the top meter of soils. This is equivalent to 52 Tg CO_2_ stored in the soils, which corresponds to 62% of the CO_2_ emissions from fossil-fuel burning by Colombia in 2014^54^. Based on average CAR estimates (122 g C_org_ m^−2^ year^−1^ over the last ~ 70 years), we estimated that seagrass meadows in the Colombian Caribbean accumulate 0.081 Tg C_org_ year^−1^ (equivalent to 0.3 Tg CO_2_ year^−1^), which corresponds to ~ 0.4% of CO_2_ emissions from fossil fuels in Colombia at 2014 rates^54^. It is important to note that these estimates are likely underestimating seagrass soil C_org_ storage in the Colombian Caribbean, owing to poor seagrass mapping in some regions and the exclusion of meadows formed by small species meadows of the genera *Syringodium*, *Halophila*, *Halodule* and *Ruppia* that occur in Colombia. However, owing to the lack of excess ^210^Pb in some meadows, further studies are required to determine whether some of the meadows studied continue to accumulate soil C_org_^40^. Indeed, carbonate burial in seagrass meadows, which is particularly important in tropical regions, likely offsets a portion of the CO_2_ sequestered through the burial of C_org_^53^. Saderne et al^55^ estimated that CaCO_3_ burial within seagrass meadows may offset ~ 30% of the net CO_2_ sequestration through photosynthesis, but acknowledged that the origin of CaCO_3_ stored within seagrass meadows is mostly derived from adjacent ecosystems rather than by calcifying organisms inhabiting or supported by seagrass ecosystems. In addition, the dissolution of CaCO_3_ linked to seagrass metabolism and associated biota, which is also common in tropical regions, results in the release of alkalinity and thereby CO_2_ removal^55,56^. Further studies are required to decipher the role of inorganic carbon processing in the overall CO_2_ budget in seagrass meadows^53,57^.

Our study demonstrates that Colombia host some of the largest seagrass soil carbon stocks per unit area globally (241 Mg C_org_ ha^−1^ in the top meter of soils), sitting between global median values (140 Mg C_org_ ha^−1^) and those of *Posidonia oceanica* meadows in the Mediterranean Sea (375 Mg C_org_ ha^−1^)^50^. This characteristic in combination with the large losses of seagrass extent over the last decades in Caribbean Coast^41,45^, places Colombia among the countries that can largely benefit from blue carbon strategies to contribute to mitigate and adapt to climate change while preserving and restoring their natural heritage and enhancing the plethora of ecosystem services that vegetated coastal ecosystems provide^2^. Colombia hosts one of the few ongoing blue carbon projects in the world, involving carbon trading by protecting 270 km^2^ of mangrove forests from deforestation^58^.

In summary, this study demonstrates that seagrass meadows along the Colombian Caribbean are hotspots for carbon sequestration and storage, provides data from a new region to a growing dataset on seagrass blue carbon and further explores differences in soil C_org_ storage based on biotic and abiotic drivers. The spatially explicit estimates of seagrass soil C_org_ stocks contribute to identify key regions for conservation and management actions (including restoration) aiming to enhance CO_2_ sequestration and/or avoid greenhouse gas emissions resulting from seagrass loss, while providing the basis for the implementation of seagrass blue carbon strategies in Colombia. The reversal of historical seagrass losses can be traded in carbon markets and in this study, we provide baseline estimates of soil C_org_ stocks and accumulation rates in Colombian seagrasses to contribute to the conservation of seagrass ecosystems through seagrass restoration strategies at the national scale.

## Materials and methods

### Study sites and sampling

This study was conducted in five regions (Alta Guajira, Media Guajira, Tayrona, San Andrés, and Cartagena Bay) along 2250 km of the Colombian Caribbean (Fig. 1). Seagrass species *T. testudinum* forms the most extensive and apparent meadows along the Colombian coast. More than 85% of seagrass extent in Colombia is located within the Guajira peninsula (562 km^2^ out of the estimated 661 km^2^)^45^.

Seagrass meadows in Alta Guajira and Media Guajira grow not deeper than 7 m water depth on sandy habitats along the shoreline, and are exposed to regular wind-driven waves, with the exception of those meadows inhabiting the few existing embayment’s. The meadows in La Guajira are not exposed to severe anthropogenic disturbances because the remoteness of the region, despite small indigenous and non-indigenous settlements may cause localized impacts^31,45^. Seagrass meadows in Tayrona National Natural Park grow in shallow sandy areas among corals and within embayment’s, and the study sites are not impacted by direct anthropogenic activities. Cartagena Bay encompasses a wide variety of seagrass habitats with different gradients of anthropogenic disturbances (mainly from industry, boating activities and dredging) and geomorphological settings (open coastlines and embayment’s) ^43^. Seagrasses in San Andrés grow on shallow soil of weathered coral and the study sites are located near the main touristic village of the island^59^. Out of the studied regions, mangrove forests are only present in Alta Guajira, Tayrona and Cartagena Bay. La Guajira is characterized by a drier tropical climate (1100 annual precipitation) compared to the other studied regions located further west of the Colombian Caribbean (ranging from 1600 mm per year in Tayrona and Cartagena Bay to 1900 mm per year in San Andrés).

A total of 14 sites across the five regions were sampled in this study (Fig. 1). Sixty-one soil cores were sampled in 0.5 to 3 m-deep meadows dominated by *T. testudinum* by manual percussion and rotation of PVC pipes (150 cm long, 65 mm inner diameter; Supplementary Information Table A). Three to twelve replicate cores were sampled within 200 m^2^ of each meadow at each site (61 cores in total). Soil compaction during coring was measured as the difference in surface soil elevation inside and outside the core^60^. The length of all cores was corrected for compaction (Supplementary Information Table A), and all results presented hereafter refer to the amended ‘uncompressed’ depths. The cores were sealed at both ends, and stored vertically at 4 °C until further processing. Seagrass plants (*T. testudinum*), mangrove (*Rhizophora mangle*) leaves, algae (*H. tuna*) and seston (> 0.7 μm) samples were collected across the study sites to determine of origin of soil C_org_ in seagrass meadows using stable carbon isotopes (Supplementary Information Table C).

### Laboratory procedures

The cores were opened longitudinally using an electric saw and cut at 1 to 15 cm-thick. The samples were dried at 60 °C until constant weight to estimate the dry weight density (g cm^−3^), and then divided in two by quartering for subsequent analyses. One set of subsamples were milled using an electric mortar and used for soil organic matter and C_org_ content analyses to estimate soil C_org_ storage, and stable carbon isotope (δ^13^C) analysis to determine the sources of soil C_org_. The other set of subsamples was used for radioisotope (^210^Pb and ^14^C) dating of the cores and soil grain-size analysis to determine distribution of particle sizes. Organic matter analyses were run in all samples from all cores sampled (N = 61), while C_org_ and grain-size analyses were only conducted in alternate samples along eight and 23 cores, respectively (Table 1).

For organic matter analyses, 4 g of milled sample was combusted at 550 °C for 4 h to estimate the proportionate loss of organic matter (loss on ignition, LOI)^61^. For the analyses of soil C_org_ and δ^13^C, 2 g of ground sample was acidified with 4% HCl to remove inorganic carbon, centrifuged (3400 revolutions per minute, for 5 min), and the supernatant with acid residues was carefully removed by pipette, avoiding resuspension. The sample was then washed with Milli-Q water, centrifuged and the supernatant removed. The residual samples were re-dried and then encapsulated for C_org_ and δ^13^C analyses using a Thermo Delta V Conflo III coupled to a Costech 4010 at the UH Hilo Analytical Laboratory (USA). The C_org_ content was calculated for the bulk (pre-acidified) samples. Macrophyte samples containing inorganic carbon and seston were acidified prior to C_org_ analysis, following the procedures above for soils. Stable carbon isotope ratios (δ^13^C) are expressed as δ values in parts per thousand (‰) relative to the Vienna PeeDee Belemnite standard. Replicate assays and standards indicated measurement errors of 0.02% for C_org_ content and 0.07‰ for δ^13^C. Sediment grain size was measured with a Coulter LS230 laser-diffraction particle analyzer at the University of Barcelona (Spain), after digestion of organic matter with 30% H_2_O_2_. The grain size data was classified in the following fractions: < 0.016 mm, > 0.016 < 0.125 mm and > 0.5 mm.

Concentrations of ^210^Pb were determined by alpha spectrometry through the measurement of its granddaughter ^210^Po, assuming radioactive equilibrium between the two radionuclides^62^. Between 150 and 300 mg of each sample were spiked with known amounts of ^209^Po and acid digested using a microwave oven. The Po isotopes were plated onto silver discs (99.99% purity) and their emissions were measured by alpha spectrometry using Passivated Implanted Planar Silicon (PIPS) detectors (CANBERRA, Mod. PD-450.18 A.M.) and silicon surface barrier detectors (ORTEC, mod. Ensemble). A selection of samples from some cores was measured for ^226^Ra by gamma spectrometry using a high-purity Ge well-type detector (CANBERRA, Mod. GCW3523) to determine the concentrations of supported ^210^Pb, used to calculate the excess ^210^Pb concentrations were calculated. The ^226^Ra concentrations were found to be comparable to those of ^210^Pb at depth in those cores, and thus the later were taken as supported ^210^Pb for the rest of the cores. The average sediment accumulation rates over the past 100 years could be estimated for six of the eleven dated sediment cores using the Constant Flux:Constant Sedimentation (CF:CS) model below the mixed surface layer when present^63^. The other cores showed either evidence of intense mixing or negligible presence of excess ^210^Pb that precluded estimating a sedimentation rate.

A total of 17 radiocarbon analyses were conducted in the bulk soil organic matter in seven cores sampled across the five regions (1–3 ^14^C analyses per core; Supplementary Information Table B) at the AMS Direct Laboratory (USA) following standard procedures^64^. The ^14^C age-depth models were produced using the R routine “Bacon” for Bayesian chronology building^65^, after calibration of ^14^C ages using the marine13 radiocarbon age calibration curve, considering a local delta R of 25 ± 8 years^66^, and assuming that the top core corresponded to the year of sampling. In two cores, the ^14^C results showed that either the core was mixed or that the samples were younger than ~ 400 years and therefore, it was not possible to estimate age-depth chronologies based on ^14^C in these cores. All ^14^C ages reported in this study are expressed as radiocarbon calibrated years.

### Numerical procedures

A linear regression between % C_org_ and % LOI (R^2^ = 0.78) was used to estimate the % C_org_ content for subsamples that were not analyzed for C_org_^5^. The density of organic carbon (g C_org_ cm^−3^) was calculated for each core slice by multiplying the sediment dry bulk density (g cm^−3^) by the C_org_ concentration (%). To allow direct comparisons among locations, the soil C_org_ stocks per unit area (cumulative stocks; Mg C_org_ ha^−1^) were standardized to 1 m-thick deposits. When sediment cores (32 out of 61) were shorter than 100 cm (65 to 90 cm), soil C_org_ stocks in the top 1 m were estimated by extrapolating the integrated values of C_org_ content (cumulative C_org_ stock; Mg C_org_ ha^−1^) linearly with depth. To validate this extrapolation approach, we assessed the correlation between extrapolated C_org_ stocks from 65 cm to 1 m and measured C_org_ stocks in 1 m soil cores across the 29 sampled cores that were equal or longer than 100 cm (r = 0.97; P < 0.001; Supporting Information Figure B). Soil C_org_ accumulation rates (expressed in g C_org_ m^−2^ year^−1^) for the last century and the last 2500 years were estimated using ^210^Pb and ^14^C age-depth models, respectively. Short-term (based on ^210^Pb) and long-term (based on ^14^C) soil C_org_ accumulation were calculated in 6 and 5 out of the 61 cores sampled, respectively, by multiplying the C_org_ inventories in 1 m-thick soil by the respective average soil accretion rate (cm year^−1^).

Pearson correlation analyses were run to assess significant relationships between soil C_org_ storage (stocks in Mg C_org_ ha^−1^ and short- and long-term accumulation rates in g C_org_ m^−2^ year^−1^) and soil δ^13^C and particles < 0.016 mm. Statistical analyses were performed using univariate General Linear Models (GLM) procedures in Statgraphics v16 software. The GLMs were run to test for significant effects of region (Alta Guajira, Media Guajira, San Andrés, Tayrona and Cartagena) on the soil organic carbon content (% C_org_), soil C_org_ stocks (Mg C_org_ ha^−1^), short- and long-term soil C_org_ accumulation rates (g C_org_ m^−2^ year^−1^), stable carbon isotope values of soil C_org_ (δ^13^C) , % < 0.016 mm, %0.016 to 0.125 mm and % > 0.5 mm (Table 3). Region was treated as fixed factor and all response variables were square-root transformed prior to analyses to meet homogeneity of variance. Normality and homoscedasticity of model residuals were assessed visually.

One-isotope, two- to three-source mixing models were run in R using simmr and rjags packages^67,68^. The isotope used in this model was δ^13^C, and the types and values of source end-members used varied across regions based on the presence/absence of potential sources and the specific values obtained for each region. Seagrass, mangrove and seston were included for Alta Guajira, Tayrona and Cartagena Bay, while only seagrass and seston were included for the Media Guajira region, and seagrass, seston and *H. tuna* for San Andrés (Supplementary Information Table C). The reference isotopic signatures (mean ± SD) used in this model comprised δ^13^C values from this study.

## Supplementary Information

Supplementary Information.
