# Supplementary material for: Seagrass blue carbon stocks and sequestration rates in the Colombian Caribbean
Source: Sci Rep. 2021 May 26;11:11067. doi: 10.1038/s41598-021-90544-5 (PMC8154905; doi:10.1038/s41598-021-90544-5)
Supplement: Supplementary file 1 — Supplementary Information. [file 41598_2021_90544_MOESM1_ESM.pdf]

Seagrass blue carbon stocks and sequestration rates in the Colombian Caribbean

Oscar Serrano[1*], Diana Isabel Gómez-López[2], Laura Sánchez-Valencia[2], Andres Acosta-Chaparro[2], Raul Navas-Camacho[2], Juan González-Corredor[2], Cristian Salinas[1], Pere Masque[1,3], Cesar Bernal[2], Núria Marbà[4]

Supplementary Information Figure A. Mean (± SE) changes in the main components along the seagrass soil cores from the regions Alta Guajira, Media Guajira, San Andrés, Tayrona, Cartagena in the Colombian Caribbean. a) Dry bulk density (g cm^{-3}); b) Organic matter content (%); c) Cumulative soil organic carbon stocks (kg C_{org} m^{-2} in 1-m thick); d) grain size particles <0.016mm (%); e) grain size particles > 0.5mm (%); f) δ^{13}C (‰); and g) soil organic carbon content (%).

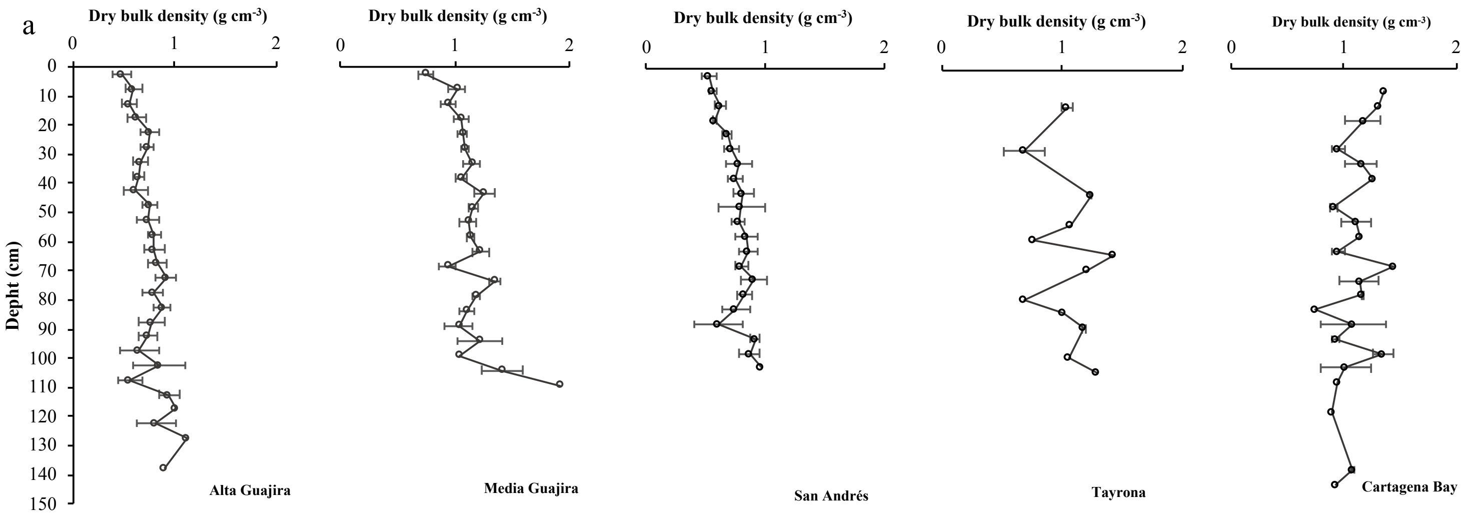

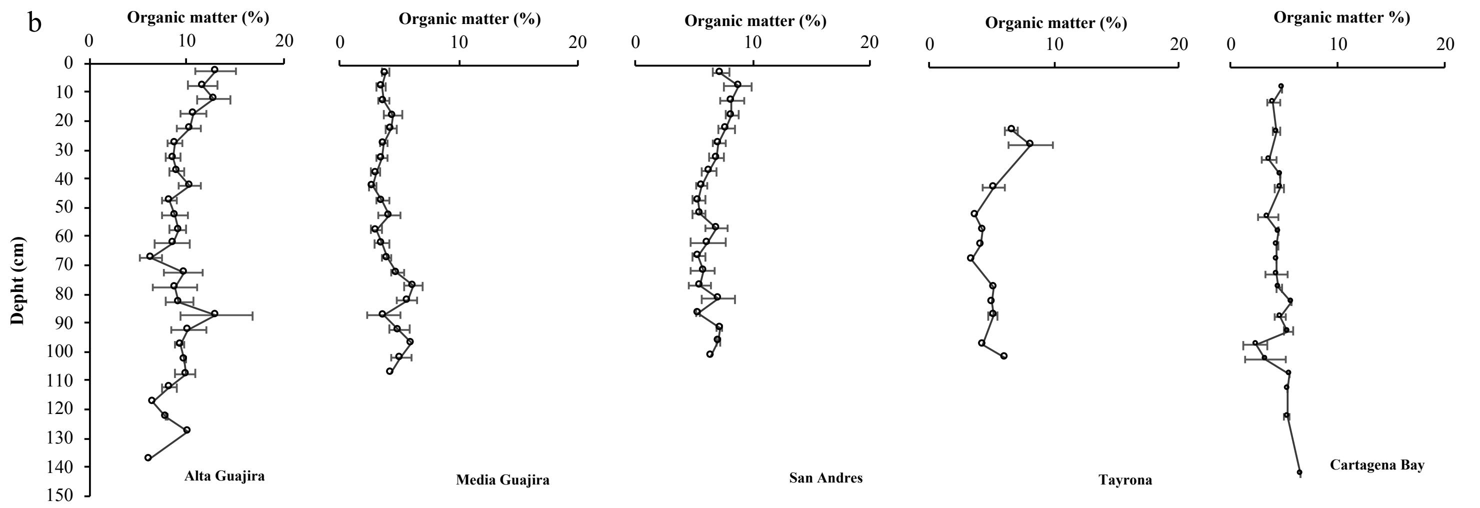

b
Organic matter (%)
Organic matter %
0
10
20
Depht (cm)
0
10
20
30
40
50
60
70
80
90
100
110
120
130
140
150
Alta Guajira
Media Guajira
San Andres
Tayrona
Cartagena Bay

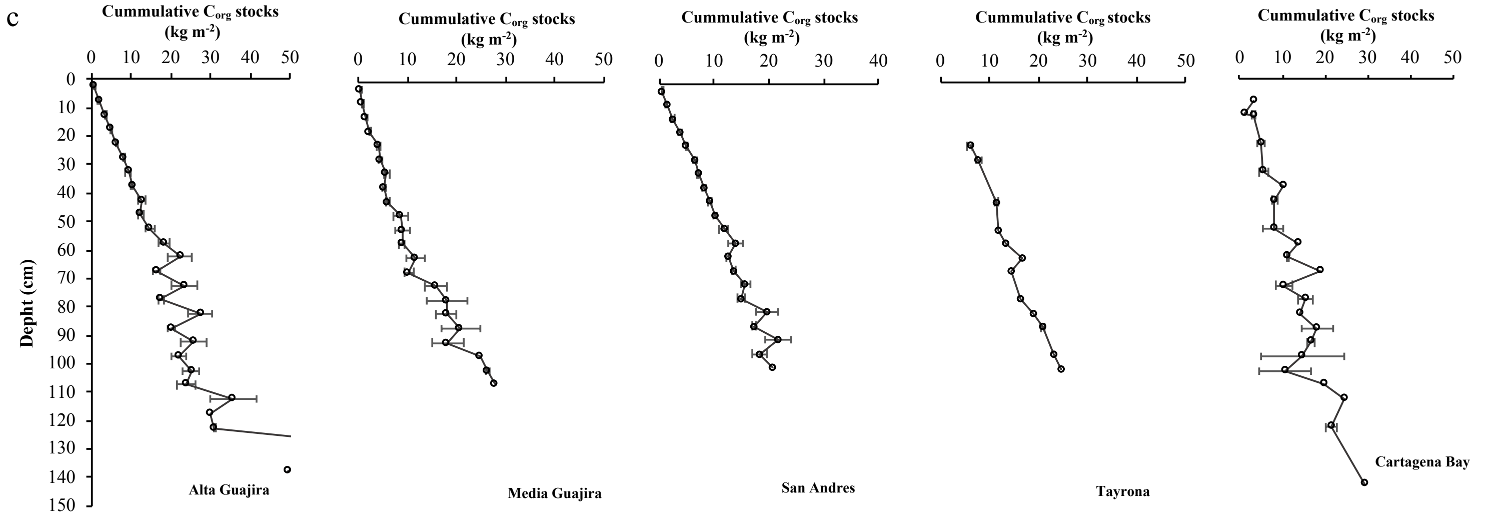

c
Cummulative C_{org} stocks ($kg\ m^{-2}$)
0
10
20
30
40
50
Depht (cm)
0
10
20
30
40
50
60
70
80
90
100
110
120
130
140
150
Alta Guajira
Media Guajira
San Andres
Tayrona
Cartagena Bay

d

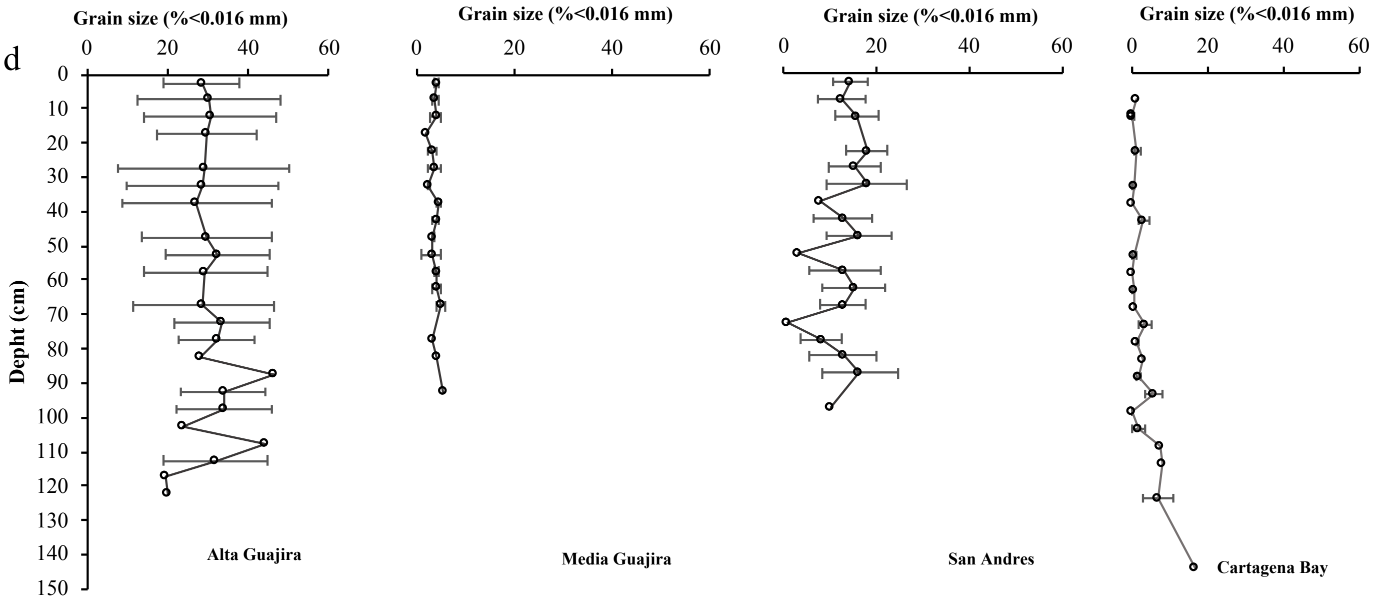
Grain size (%<0.016 mm)
0
20
40
60
Depht (cm)
0
10
20
30
40
50
60
70
80
90
100
110
120
130
140
150
Alta Guajira
Media Guajira
San Andres
Cartagena Bay

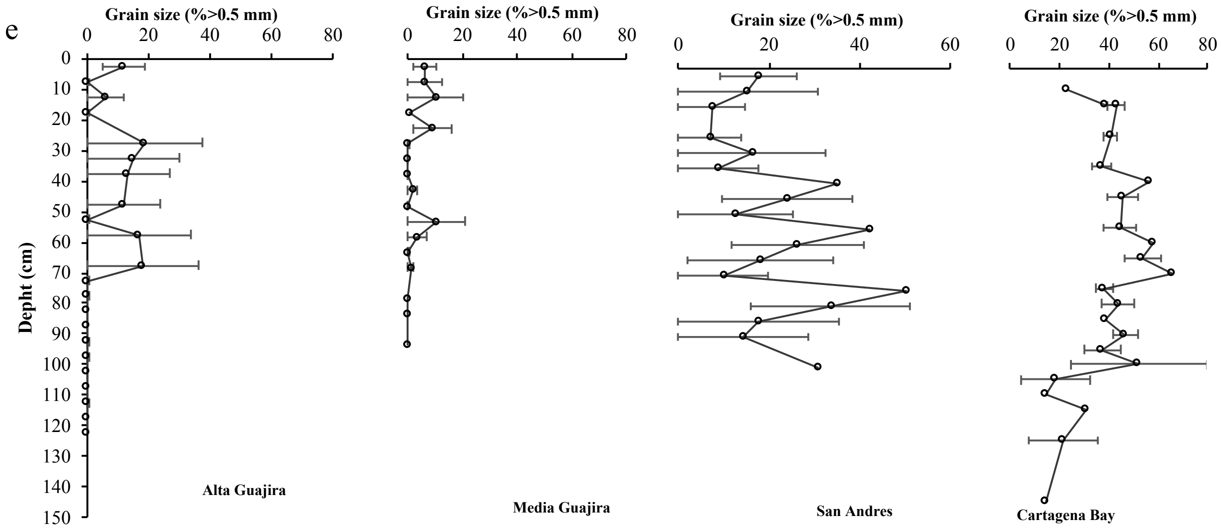
e
Grain size (%>0.5 mm)
Depht (cm)
Alta Guajira
Media Guajira
San Andres
Cartagena Bay

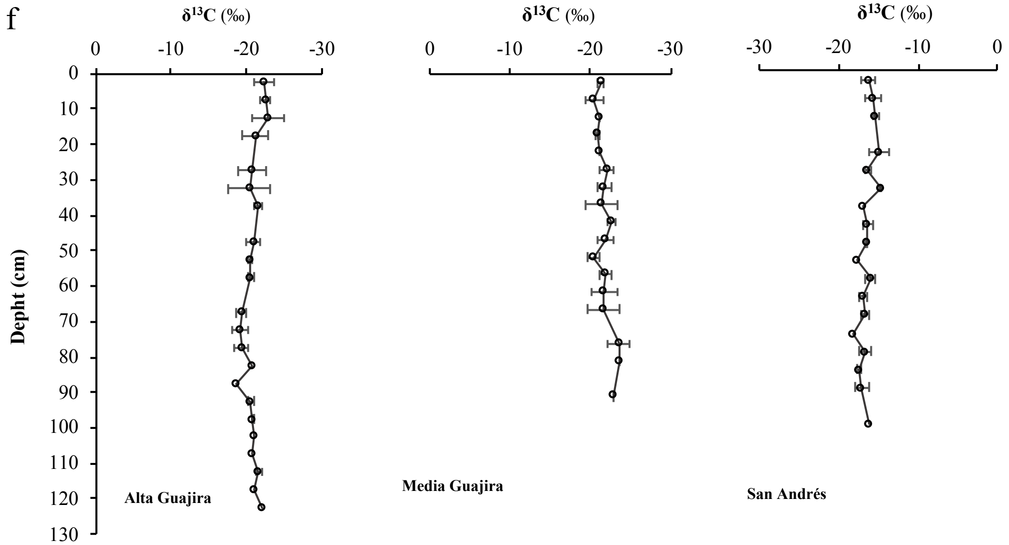
f
δ13C (‰)
Depht (cm)
Alta Guajira
Media Guajira
San Andrés

g

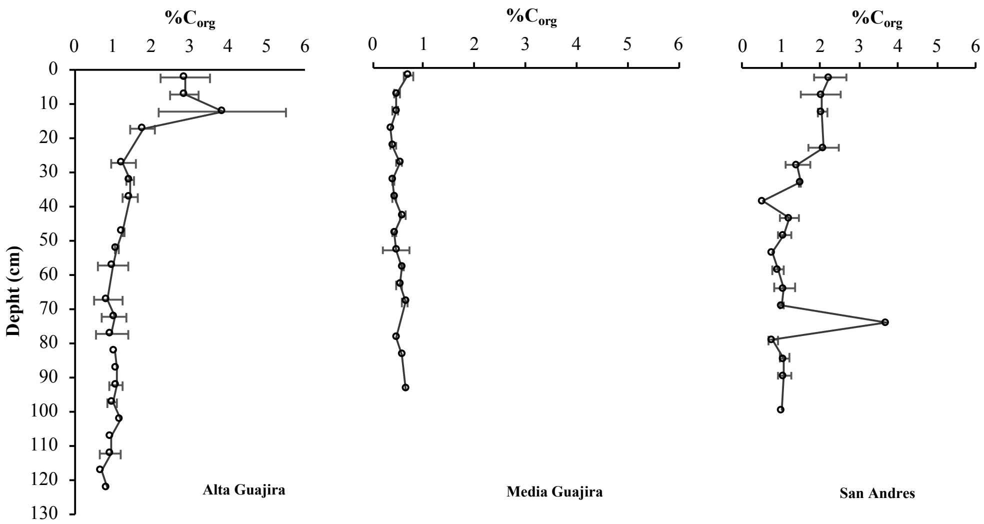
%Corg
0 1 2 3 4 5 6
Depht (cm)
0
10
20
30
40
50
60
70
80
90
100
110
120
130
Alta Guajira
Media Guajira
San Andres

Supplementary Information Figure B. Relationship between soil organic carbon stocks measured at 1 m (Mg C_{org} ha^{-1}) and extrapolated from 0.65 m to 1 m from cores ≥ 1 m-thick.

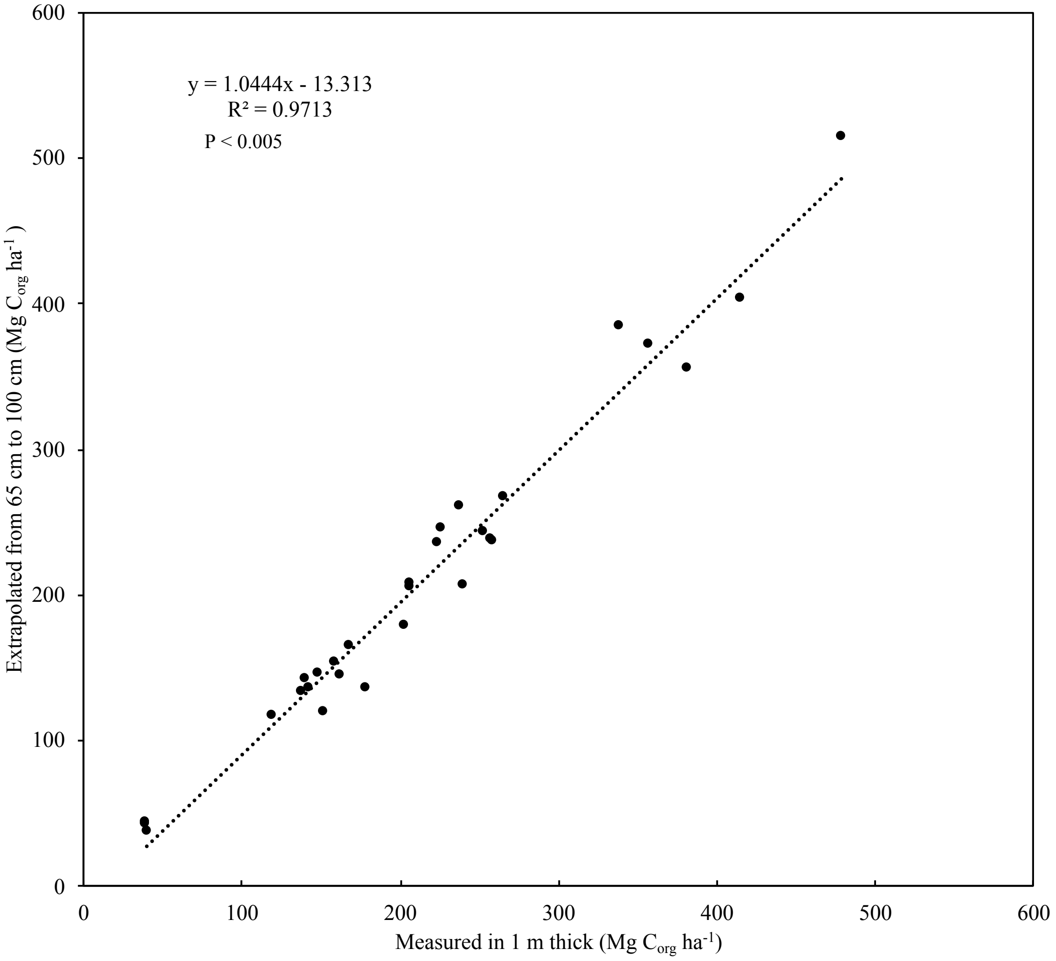

Supplementary Information Table A. Details of the seagrass cores sampled, including the % compression during coring operations. Core length corrected for compression (cm decompressed).

**Core ID**	**Region**	**Location**	**Latitude**	**Longitude**	**Core length (cm comp.)**	**Core length (cm decomp.)**	**Compression (%)**
SABHK1	San Andrés	Bahía Hooker	12° 33' 46.1" N	81° 42' 1.22" W	73	100	38
SABHK2			12° 33' 46.1" N	81° 42' 1.22" W	69	94	36
SABHK3			12° 33' 46.1" N	81° 42'1.22" W	72	96	33
SACC1		Cotton Cay	12° 11' 50.5" N	72° 08' 56.7" W	63	87	38
SACC2			12° 11' 50.5" N	72° 08' 56.7" W	54	73	34
SACC3			12° 12' 10.88" N	72° 09' 7.27" W	48	65	39
SAMA1		Mar Azul	12° 12' 10.88" N	72° 09' 7.27" W	58	83	44
SAMA2			12° 12' 10.88" N	72° 09' 7.27" W	60	84	40
SAMA3			12° 33' 31.41" N	81° 42' 4.82" W	40	56	60
AGBH1	Alta Guajira	Bahía Hondita	12° 33' 31.41" N	81° 42' 4.82" W	66	93	41
AGBH2			12° 33' 31.41" N	81° 42' 4.82" W	60	85	51
AGBH3			11° 19' 3.53" N	74° 08' 1.87" W	95	124	30
AGBH4			11° 19' 14.44" N	74° 07' 35.41" W	90	127	19
AGHB5			11° 19' 14.44" N	74° 07' 35.41" W	96	136	21
AGBH6			12° 03' 51.7" N	72° 09' 27.0" W	86	114	33
AGBH7			11° 19' 3.53" N	74° 08' 1.87" W	58	82	52
AGBH8			12° 03' 51.7" N	72° 09' 27.0" W	50	71	59

AGBP1		Bahía Portete	12° 03' 51.7" N	72° 09' 27.0" W	40	65	61
AGBHP2			11° 56' 15.6" N	72° 12' 49.6" W	45	70	56
AGBHP3			11° 56' 15.6" N	72° 12' 49.6" W	48	73	53
AGBHP4			11° 56' 15.6" N	72° 12' 49.6" W	38	64	67
AGBHP5			11° 56' 14.24" N	72° 12' 50.84" W	41	66	61
AGCV1		Cabo de la Vela	11° 56' 14.24" N	72° 12' 50.84" W	71	94	33
AGCV2			11° 56' 14.24" N	72° 12' 50.84" W	51	70	36
AGCV3			11° 56' 08.5" N	72° 12' 52.6" W	30	45	51
AGCV4			11° 56' 08.5" N	72° 12' 52.6" W	46	65	40
AGCV5			11° 56' 08.5" N	72° 12' 52.6" W	38	61	61
MGCZ1	Media Guajira	Carrizal	10° 23' 52" N	75° 32' 31" W	76	92	21
MGCZ2			10° 15' 53" N	75° 35' 32" W	67	83	24
MGCZ3			10° 15' 53" N	75° 35' 32" W	90	107	19
MGCZ4			10° 19' 12" N	75° 35' 17" W	47	69	48
MGCZ5			10° 15' 53" N	75° 35' 32" W	47	69	46
MGCZ6			10° 19' 12" N	75° 35' 17" W	46	68	47
MGCZ7			10° 19' 12" N	75° 35' 17" W	61	87	43
MGCZ8			10° 23' 52" N	75° 32' 31" W	65	84	30
MGCZ9			10° 23' 52" N	75° 32' 31" W	70	83	19
MGCZ10			10° 22' 46" N	75° 34' 22" W	48	72	50
MGCZ11			10° 22' 46" N	75° 34' 22" W	39	62	59
MGCZ12			10° 22' 46" N	75° 34' 22" W	40	62	55
MGMC1		Musichi	10° 21' 57" N	75° 32' 50" W	65	91	39
MGMC2			10° 21' 57" N	75° 32' 50" W	59	84	43
MGMC3			10° 21' 57" N	75° 32' 50" W	68	89	31

SMCH1	Tayrona	Bahía Chengue	12° 24' 56.9" N	71° 42' 45.1" W	52	76	46
SMCH2			12° 24' 56.9" N	71° 42' 45.1" W	62	83	34
SMCH3			12° 25' 04.8" N	71° 42' 28.9" W	92	103	12
SMCH4			12° 25' 04.8" N	71° 42' 28.9" W	90	97	8
CLV1	Cartagena	La Virgen	12° 25' 04.8" N	71° 42' 28.9" W	81	114	41
CLV2			12° 25' 45.1" N	71° 41' 37.6" W	67	95	41
CLV3			12° 25' 45.1" N	71° 41' 37.6" W	70	102	45
CTTB1		Tierra Bomba	12° 25' 45.1" N	71° 41' 37.6" W	91	117	28
CTTB2			12° 34' 31.52" N	81° 41' 38.09" W	120	156	30
CTTB3			12° 34' 31.52" N	81° 41' 38.09" W	109	133	22
CTPA1		Punta Arena	12° 34' 31.52" N	81° 41' 38.09" W	80	110	38
CTPA2			11° 44' 56.62" N	72° 35' 38.76" W	100	125	25
CTPA3			11° 44' 56.62" N	72° 35' 38.76" W	86	112	31
CTBC1		Bahia Cocon	11° 44' 56.62" N	72° 35' 38.76" W	79	109	38
CTBC2			12° 10' 30.12" N	71° 57' 37.83" W	76	86	13
CTBC3			12° 10' 30.12" N	71° 57' 37.83" W	108	108	0
CTBG1		Bocagrande	12° 10' 30.12" N	71° 57' 37.83" W	114	129	13
CTBG2			12° 09' 53.6" N	71° 58' 5.01" W	110	117	7
CTBG3			12° 09' 53.6" N	71° 58' 5.01" W	88	92	4

Supplementary Information Table B. Radiocarbon results of the soil organic carbon in seagrass cores sampled in the Colombian Caribbean.

DirectAMS code	Core ID	Region	Location	Depth (cm decompressed)	Radiocarbon age	
					BP	1sigma error
D-AMS 031357	SABH1	San Andrés	Bahía Hooker	45	400	26
D-AMS 031358	SABH1	San Andrés	Bahía Hooker	66	414	27
D-AMS 031359	SABH1	San Andrés	Bahía Hooker	93	479	25
D-AMS 031360	SACC1	San Andrés	Cotton Cay	47	*Modern*	
D-AMS 031361	SACC1	San Andrés	Cotton Cay	59	81	26
D-AMS 031362	SACC1	San Andrés	Cotton Cay	76	401	24
D-AMS 031364	MGCZ10	Media Guajira	Carrizal	55	*1304*	*27*
D-AMS 031365	MGCZ10	Media Guajira	Carrizal	64	*972*	*25*
D-AMS 031366	MGCZ4	Media Guajira	Carrizal	48	87	26
D-AMS 031367	MGCZ4	Media Guajira	Carrizal	55	281	27
D-AMS 031368	MGCZ4	Media Guajira	Carrizal	63	315	24
D-AMS 031371	SAMA1	San Andrés	Mar Azul	79	517	31
D-AMS 031373	MGMC1	Media Guajira	Musichi	66	2184	40
D-AMS 031374	MGMC1	Media Guajira	Musichi	80	3017	32
D-AMS 031375	AGBH3	Alta Guajira	Bahía Hondita	42	1430	29
D-AMS 031376	AGBH3	Alta Guajira	Bahía Hondita	75	2333	32
D-AMS 031377	AGBH3	Alta Guajira	Bahía Hondita	114	2560	41

Supplementary Information Table C. Mean (± SD) stable carbon isotopes ($\delta^{13}C$, (‰) in seagrass, mangrove, seston and *Halimeda tuna* (mean and SD) across the regions studied in the Colombian Caribbean.

Region	Source	$\delta^{13}C$ (‰) mean	SD
Alta Guajira	Seagrass	-10.7	1.1
	Mangrove	-29.0	0.1
	Seston	-30.6	0.5
Media Guajira	Seagrass	-9.1	0.5
	Seston	-27.8	3.7
San Andrés	Seagrass	-8.3	0.5
	Seston	-25.0	4.1
	Halimeda tuna	-17.0	0.1
Tayrona	Seagrass	-8.9	1.2
	Mangrove	-29.0	0.1
	Seston	-28.9	3.1
Cartagena Bay	Seagrass	-8.9	1.2
	Mangrove	-29.0	0.1
	Seston	-28.9	3.1
